# Treatments for different types of ERCP-related Stapfer IV injury: a case report and literature review

**DOI:** 10.3389/fmed.2025.1549795

**Published:** 2025-04-30

**Authors:** Hao Liang, Min Yang, Yuan-Jun Liu

**Affiliations:** Department of Hepatobiliary Surgery, Suining Central Hospital, Suining, Sichuan, China

**Keywords:** endoscopic retrograde cholangiopancreatography, infections, Stapfer IV injury, cholecystitis, pneumothorax, conservative treatment

## Abstract

**Background:**

As the endoscopic retrograde cholangiopancreatography (ERCP) is commonly used, some rare ERCP-related perforation cases have garnered attention. This study aims to report a rare case of Stapfer IV injury accompanied by severe infections and review the appropriate treatment methods for patients with different types of Stapfer IV injury.

**Case summary:**

A female patient received ERCP treatments, but she soon presented with massive diffuse abdominal and thoracic gas accompanied by severe infections. In the following upper gastrointestinal radiography examination, no signs of gastrointestinal perforations were observed. After receiving antibiotic treatments and percutaneous transhepatic gallbladder drainage therapy, the patient recovered and was discharged. Finally, she was diagnosed with Stapfer IV injury and cholecystitis.

**Conclusion:**

Simple Stapfer IV injury was not a true perforation, and the conservative treatment was feasible. When a severe infection occurs in a patient with Stapfer IV injury, the other complications of ERCP procedures, such as cholecystitis, should be taken into consideration. Interventions for the source of infections, not just for the Stapfer IV injury, might be effective.

## 1 Background

Endoscopic retrograde cholangiopancreatography (ERCP), endoscopic sphincterotomy (EST), and endoscopic papillary large-balloon dilation (EPLBD) have increasingly been recommended for the treatment of common bile duct stones (1). However, some severe complications, such as hemorrhage, pancreatitis, cholangitis, and perforation, cannot always be avoided in those procedures as the occurrence of complications was primarily related to the inexperienced operators in endoscopic techniques (2).

Perforation, different from other ERCP-related complications, was an unusual and complex issue. The mortality of patients with ERCP-related perforation was fairly high. Approximately 20% (75/376) of patients died within 90 days after being diagnosed with ERCP-related perforations (3). The prognosis of those patients may be influenced by different types of perforations and the time of performing surgical interventions (4). In clinical practice, the Stapfer classification (I, II, III, and IV types) of ERCP-related perforations has been posed and widely used for a long time (5). However, there are different perspectives on the indications of surgical intervention for the patient with perforations.

In patients with ERCP-related perforations, the incidence of Stapfer IV injury was relatively low (9/63, 14.3%), and similar cases were rarely reported (4). When those patients presented with severe infections, selecting appropriate time and proper intervention methods, including surgical treatments, were critical for improving prognosis (6, 7). However, few studies reported treating patients with Stapfer IV injury accompanied by severe infections.

This study reported on a patient with both severe Stapfer IV injury and infections and reviewed the treatment of different types of Stapfer IV injury.

## 2 Case report

A 36-year-old woman was admitted to the Department of Hepatobiliary Surgery due to her intermittent upper abdominal pain. None of the special medical history was recorded. During the examination, the patient was cooperative, but the tenderness upon abdominal palpation was noted. No jaundice of the sclera was observed.

**Figure 1:**
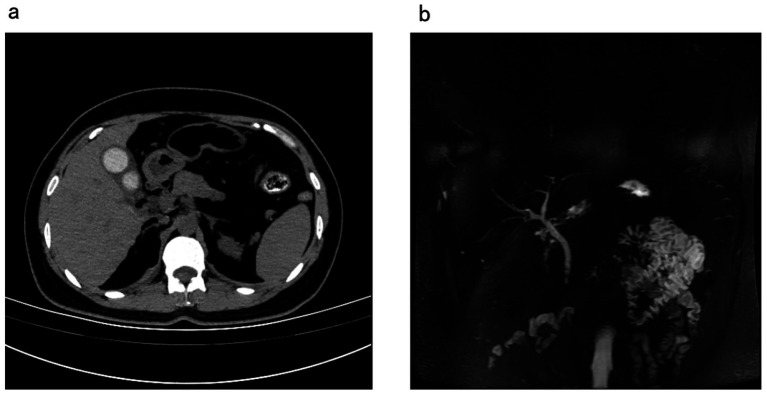
Patient’s abdomen MRI and MRCP before the ERCP treatment; **(a)** signs of cholecystitis and gallstones; **(b)** stone signs in the common bile duct. ERCP, endoscopic retrograde cholangiopancreatography; MRCP, magnetic resonance cholangiopancreatography; MRI, magnetic resonance imaging.

The results of magnetic resonance imaging (MRI) of abdomen and magnetic resonance cholangiopancreatography (MRCP) showed the following: (1) there were some signs of gallstones and cholecystitis (Figure 1a); and (2) some small low-signal nodules were observed in the common bile duct (CBD), possibly stones (Figure 1b). The white blood cell (WBC) count was 8.1 × 10^^9^/L (reference range: 3.5 to 9.3). Her blood total bilirubin level was 10.6 μmol/L (reference range: 5.1 to 28.0).

Initially, she received treatment for ERCP, EST, and EPLBD. The sediment-like stones were picked out from the CBD, and the previously observed filling defects in the ERCP examination disappeared. However, the placement of the naso-biliary tube was unsuccessful. In the procedure, no obvious perforations were observed. Although she had received those treatments, the patient still complained of upper abdominal pain.

**Figure 2:**
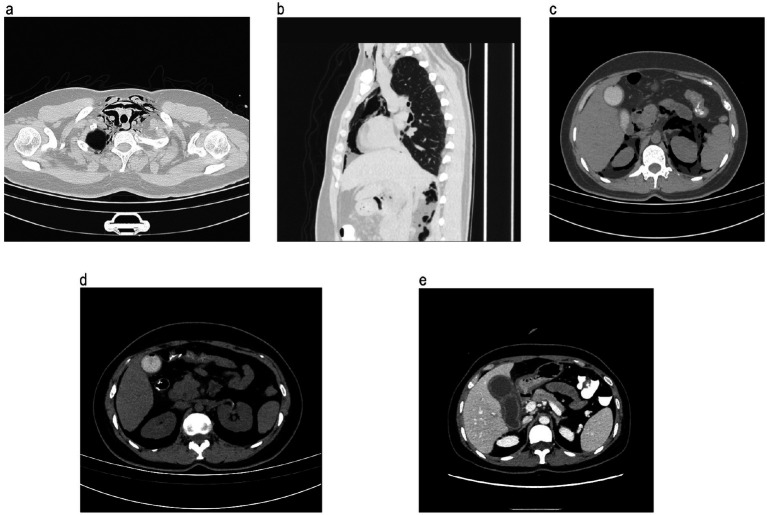
Patient’s computed tomography of the abdomen and chest after the ERCP treatment; **(a)** gas density shadows in the soft tissue in front of the cervical vertebra; **(b)** gas density shadows in the mediastinum and bilateral chest subcutaneous; **(c)**, gas density shadows in the upper abdominal cavity; **(d)** gas density shadows in the gallbladder; and **(e)** the volume and density of gallbladder obviously increased within some low-density shadows. ERCP, endoscopic retrograde cholangiopancreatography.

Two days later, the symptom of upper abdominal pain persisted. Importantly, the subcutaneous emphysema was palpated in her neck and upper chest area on examination. Then, the computed tomography (CT) results showed that there were numerous gas density shadows in her bilateral neck, submaxillary region, right maxillofacial area, and retropharyngeal and parapharyngeal space, as well as in the soft tissue in front of the cervical vertebra, mediastinum, bilateral chest subcutaneous, and upper abdominal cavity (Figures 2a–c). The volume and density of the gallbladder obviously increased, with some low-density shadows mixed in it (Figures 2d,e). At this time, the WBC count was normal, 9.2 × 10^^9^/L, but the neutrophils count increased, 8.05 × 10^^9^/L (reference range, 1.8 to 6.3). The next day, the WBC count suddenly increased to 24.7 × 10^^9^/L, and the neutrophil count was high at 22.34 × 10^^9^/L. In addition, the hypersensitivity C-reactive protein (hs-CRP) level was very high, 126.18 mg/L (reference range, 0 to 10), and the procalcitonin level reached 26.1 ng/mL (reference range <0.5).

This patient was diagnosed with ERCP-related perforation accompanied by severe infections. The relationships between perforation and severe infections were nebulous. Choosing either the conservative treatment or surgical intervention had numerous risks, which may be a critical life-threatening decision. As for potential reasons of the massive abdominal and thoracic gas along with severe infections, the simple severe infections in the retroperitoneum, perforation-related pancreatic fistula, intestinal fistula, and bile leak were taken into consideration. The two events, namely, severe infections and massive abdominal and thoracic gas, might be unrelated. Therefore, the multi-disciplinary treatment was conducted.

**Figure 3:**
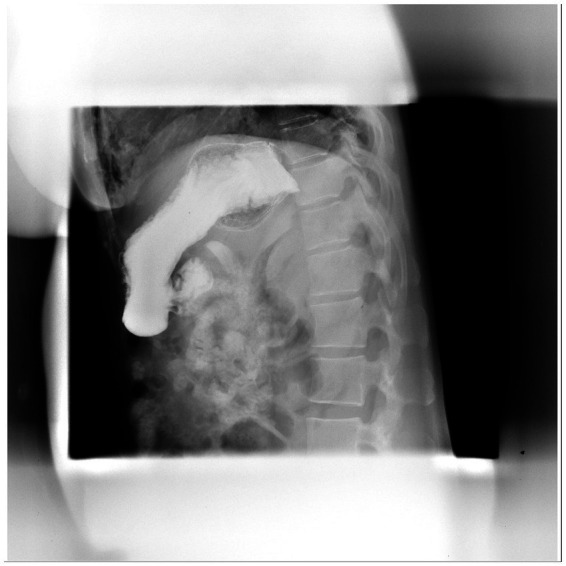
No signs of gastrointestinal perforation in the upper gastrointestinal radiography examination.

The next day, the patient received conservative treatment, including antibiotic treatment (Sulperazon) and gastrointestinal decompression therapy. Meanwhile, the results of the following upper gastrointestinal radiography examination did not indicate any signs of gastrointestinal perforations (Figure 3). Then, given the severe cholecystitis, the patient received the percutaneous transhepatic gallbladder drainage therapy (PTGD), and the result of bile culture showed the infection of *Morganella morganii*. Fortunately, this microorganism was sensitive to the use of antibiotic treatment.

Two days later, the results of the CT examination showed that gas density shadows in the patient’s bilateral neck, submaxillary, right maxillofacial, and retropharyngeal and parapharyngeal space, as well as in the soft tissue in front of cervical vertebra, mediastinum, bilateral chest subcutaneous, and upper abdominal cavity reduced in some degree, compared to the previous examination. There was no new extra effusion in those tissues. Importantly, the level of hs-CRP and procalcitonin of this patient dropped to 61.02 mg/L and 15.18 ng/mL, respectively.

**Figure 4:**
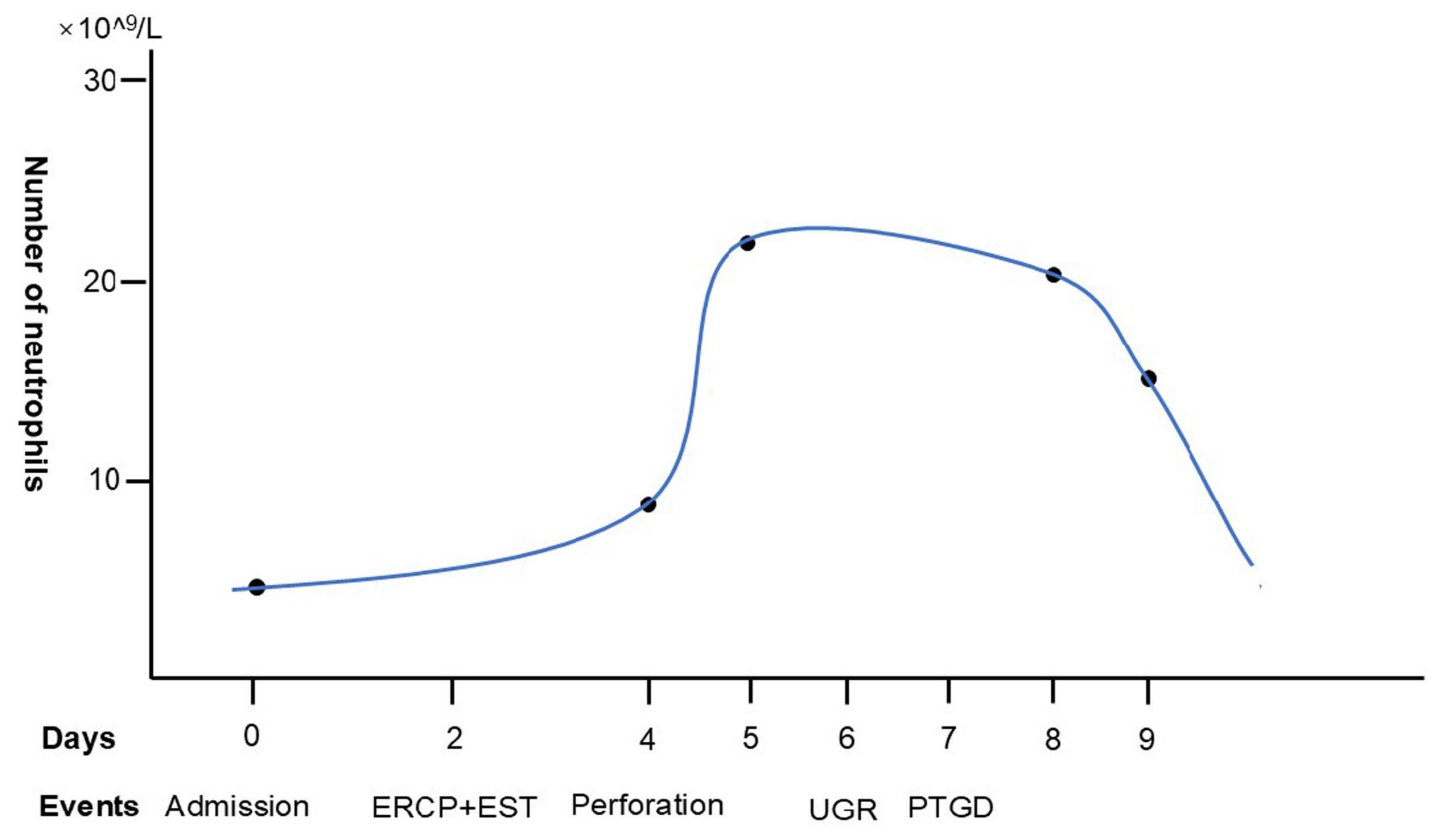
Number of blood neutrophil count of the patient in the whole diagnosis and treatment process. ERCP, endoscopic retrograde cholangiopancreatography; EST, endoscopic sphincterotomy; UGR, upper gastrointestinal radiography; PTGD, percutaneous transhepatic gallbladder drainage.

Five days following the PTGD therapy, the abdomen tenderness disappeared, and all of the inflammation markers, including the WBC counts, remained within normal ranges (Figure 4). Ultimately, the patient underwent laparoscopic cholecystectomy and was discharged safely.

## 3 Discussion and literature review

A rare case was reported of a patient presenting massive diffuse gas in many tissues accompanied by severe infections after undergoing the ERCP treatment. Based on the PTGD and antibiotics therapy, the patient was relieved from the terrible injury without receiving the surgical intervention.

Perforation was a quite unusual ERCP-related complication. The incidence of ERCP-related perforation was always lower than 3% (3, 8–10). A few patients were diagnosed with perforation after 24 h of the ERCP procedures (9/75, 12.0%) (11). The classical four classes of ERCP-related perforations (Stapfer types) were first posed in the 2000 years (5), and this classification was widely used due to its great value in guiding treatments. The number of Stapfer IV injuries (retroperitoneal air alone) was small (9/63, 14.28%) in the patients with perforations (4). However, in the large part of patients with perforations, the multi-organ failure was the high-risk factor of mortality, including infections (9/62, 15.0%) (3). Thus, in clinical practice, patients with complex Stapfer IV injuries accompanied by severe infections should be closely cared.

**Figure 5:**
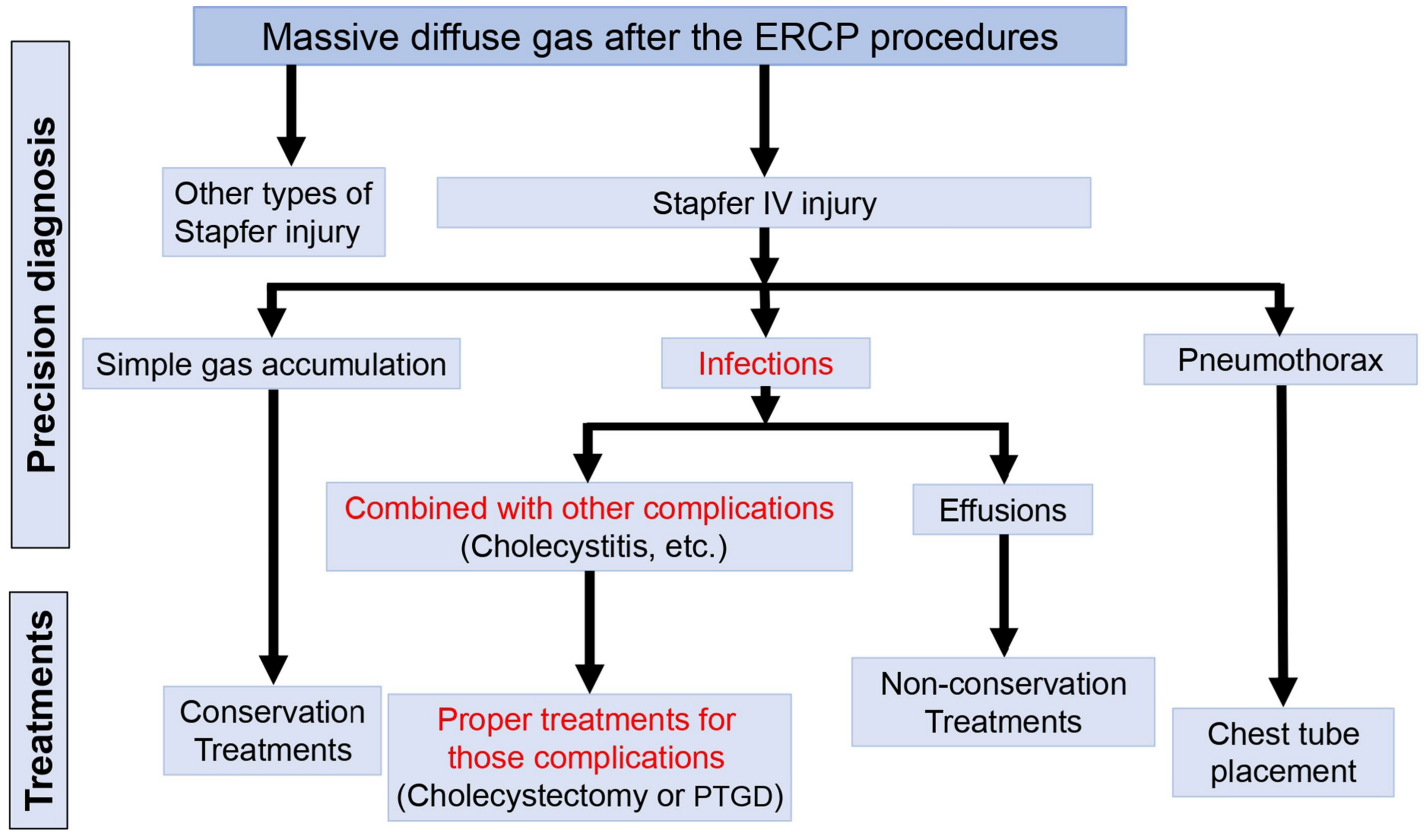
Flow gram of treatments for different types of Stapfer IV injury. ERCP, endoscopic retrograde cholangiopancreatography; PTGD, percutaneous transhepatic gallbladder drainage.

In the following parts, treatments of different types of Stapfer IV injury were reviewed (Figure 5).

### 3.1 Treatments for simple Stapfer IV injury

Simple Stapfer IV injury may not be a true perforation (5). In our case, the negative result of the upper gastrointestinal radiography examination, which failed to identify the perforation location, supported this perspective. The probable mechanism of Stapfer IV injury involved the numerous compressed airs along the perineural and perivascular sheath flight into the mediastinum (12). Another possible mechanism was that prolonged air insufflation caused air to dissect through the retroperitoneum and peritoneal cavity into the pleural space, mediastinum, and subcutaneous tissue of neck. Placement of a naso-biliary tube after the ERCP procedure or avoiding numerous and prolonged compressed air insufflation in the whole ERCP procedure might reduce the occurrence of simple Stapfer IV injury (13). According to the above pathological mechanism, conservative management was appropriate for the simple Stapfer IV injury (14). Stapfer IV injury may not be an absolute indication for surgical exploration (15).

### 3.2 Treatments for Stapfer IV injury along with pneumothorax

However, some special interventions for complex Stapfer IV injury must be considered. Some patients experienced Stapfer IV injury accompanied by pneumothorax (16–18). The chest tube placement treatment for that circumstance could be effective (19–21). The previous study showed that the massive bilateral chest subcutaneous gas was one of the surgical indications for this type of Stapfer IV injury (5).

### 3.3 Treatments for Stapfer IV injury along with infections

Under the background of ERCP-related perforation, the patient with severe infections must be noted. Previous studies reported that the sepsis may indicate surgical interventions (22). Antibiotic therapy and intensive care were necessary. However, investigating the source of infections and assessing the correlation between perforation and infections were two key steps to successful treatment. The presence of effusion collection in the retroperitoneal or peritoneum could have indicated the potential relationship between perforation and infections. In these circumstances, surgical interventions should be considered (23).

When patients presented with Stapfer IV injury and infections without effusion collection, other complications of ERCP procedures might exist, including cholecystitis. The rapid occurrence of cholecystitis after the ERCP procedure was rare (24). The coexistence of severe cholecystitis and Stapfer IV injury is quite rare, and there is no standard treatment scheme for this particular case currently. Different from the positive surgical exploring indications in previous studies (5), patients with severe cholecystitis after the ERCP-related perforation could be treated successfully without surgical interventions. In addition, surgical interventions could be considered for treating cholecystitis or other complications of ERCP procedures, not just for the Stapfer IV injury.

### 3.4 Potential bacterial translocation in Stapfer IV injury

*Morganella morganii*, as a part of the normal flora in the intestinal tracts (25), was not a common pathogen of cholangitis. It was rarely identified in bile cultures from the gallbladder. Infection of *Morganella morganii* in the gallbladder might be associated with the Stapfer IV injury as a few gases were detected in the patient’s gallbladder. This sign may reveal that compressed air in the Stapfer IV injury might carry some normal flora to other parts of the body, leading to life-threatening infections. However, none of these types of cases have been reported in the previous literature. In this situation, surgical interventions could be operated for the cholangitis. To mitigate the risk of infection and Stapfer IV injury, adherence to standardized ERCP operation was essential. In addition, preventive usage of antibiotics could reduce the risk of infections in some high-risk patients (26).

## 4 Conclusion

Conservative treatment for simple Stapfer IV injury was feasible and reliable. Stapfer IV injury patients with pneumothorax could recover through the placement of a chest tube. When severe infection occurs in patients with Stapfer IV injury, other complications of ERCP procedures, cholecystitis, should be taken into consideration. Surgical interventions for the source of infections, not just for the Stapfer IV injury, might be effective.

## Data Availability

The original contributions presented in the study are included in the article/supplementary material, further inquiries can be directed to the corresponding author.
